# Human genetic diversity across South Asian populations: A systematic review and meta-analysis

**DOI:** 10.1097/MD.0000000000044147

**Published:** 2025-08-29

**Authors:** Shafee Ur Rehman, Ghulam H. Abbas

**Affiliations:** a Faculty of Medicine, Ala-Too International University, Tunguch, Bishkek, Kyrgyzstan.

**Keywords:** Fixation Index, genetic diversity, genome-wide association studies, population structure, South Asia

## Abstract

**Background:**

South Asia comprises genetically diverse populations because its populations have experienced multiple migrations combined with endogamy and isolation throughout history. Research on large-scale genetic variation patterns in this region remains scarce. This study aims to perform both a systematic review and meta-analysis of research about genetic diversity in South Asian populations.

**Methods:**

The PubMed database yielded 3798 studies within the time frame from 2010 to 2025. The analysis included 57 studies that fulfilled the required criteria after initial screening. The random-effects model processed pooled F_ST values together with heterozygosity estimates and allele frequency variation across ethnic subgroups.

**Results:**

The genetic differentiation (F_ST) measurements between significant South Asian groups extended from 0.02 to 0.15. The homozygosity levels were significantly higher in tribal populations (mean runs of homozygosity = 0.38) than in caste groups. The combined F_ST value reached 0.072 with a 95% confidence interval ranging from 0.061 to 0.084. The highest heterozygosity values existed among North Indian speakers of the Indo-European language, while isolated tribal populations showed the lowest heterozygosity levels.

**Conclusion:**

The genetic structure of South Asia extends deep into its population because of geographical barriers as well as linguistic and social organization systems. The diverse genetic makeup of populations affects both disease risk profiles and precision medical approaches for individual groups.

## 1. Introduction

South Asia represents a highly diverse genetic territory because its people have been influenced by historical migrations as well as social organization systems and different geographical regions.^[1–3]^ Throughout millennia the region has been home to successive human migrations that started with hunter-gatherers followed by Neolithic farmers from the Fertile Crescent and eventually Indo-European-speaking pastoralists.^[3–5]^ The migrations and prolonged endogamous practices especially among caste and tribal communities formed an exceptional genetic pattern. Population isolation and differentiation occurred because of geographical barriers including the Himalayas and Indian Ocean thus creating different genetic substructures among small geographic areas.^[6,7]^ South Asia stands as one of the most genetically diverse regions yet scientists know very little about its genetic makeup because of inadequate representation in global genomic studies.^[8]^

The majority of large genetic research projects have analyzed European and East Asian populations, but they fail to provide adequate information about South Asian ancestry for personalized medicine applications.^[9]^ The insufficient representation of South Asian populations creates obstacles for researching both genetic disorders and complex diseases that demand unique therapeutic approaches and risk factor analyses in South Asian populations.^[10]^ Scientific and biomedical progress demands addressing this knowledge gap because South Asia contains approximately 25% of global population numbers.^[11,12]^ Recent research has exposed the detailed ancestral genetic splits which separate Indo-European from Dravidian speakers while showing more detailed genetic divisions between caste groups and tribes in South Asia.^[13]^ The combination of social hierarchies with endogamy has allowed genetic distinctions to persist across multiple generations.

The genetic traits found in isolated tribal groups and highland populations contain rare genetic material that scientists believe could explain human adaptation mechanisms and disease resistance abilities.^[14,15]^ Many populations in this region lack complete genomic mapping because scientists have not studied or adequately represented them in genetic databases.^[16,17]^ Research combining existing genetic studies across various South Asian populations allows scientists to identify ancestral connections and distinct genetic characteristics which the analysis uncovers through data synthesis.^[18]^ The research will enhance scientists’ comprehension of how historical migrations and social and environmental elements have formed the genetic structure of the region. Future research needs to establish inclusive sampling methods together with collaborative studies which will produce fair global genomics representation for scientific advancement and public health programs in South Asia and worldwide.

## 2. Methods

### 2.1. Ethical review

The research consists of a meta-analysis that relies solely on peer-reviewed literature and publicly available datasets. The study did not require new human participant data collection so ethical approval and informed consent were not needed. The original studies included in this research received ethical approval from institutional review boards or ethics committees when they were first published.

### 2.2. Search strategy

A research of literature was conducted using 4 electronic databases: PubMed, Scopus, Web of Science, and Google Scholar, covering studies published between January 2010 and March 2025. The following keywords and Boolean operators were used: (“genetic diversity” OR “population structure” OR “genomic variation”) AND (“South Asia” OR “India” OR “Pakistan” OR “Bangladesh” OR “Sri Lanka” OR “Nepal”) AND (“whole genome sequencing” OR “SNP array” OR “Y-chromosome” OR “mtDNA”). Searches were limited to English-language studies. The title, abstract, and keywords fields were searched in each database.

### 2.3. Inclusion and exclusion criteria

Studies were included if they met the following criteria:

- Studies conducted in South Asian populations.
- Peer-reviewed original research articles.
- Used genome-wide, exome-based, single nucleotide polymorphism (SNP) array, or targeted sequencing approaches.
- Reported quantitative data (e.g., *F*_ST_, heterozygosity, allele frequencies, principal component analysis results).
- Published between 2010 and 2025.

### 2.4. Exclusion criteria

- Studies focused on non-South Asian populations.
- Studies lacking quantitative metrics for genetic diversity.
- Case reports or studies with sample size <30.

Both abstracts and full texts were evaluated, including any supplementary materials. Two independent reviewers conducted the search and screening process. Disagreements were resolved by discussion or consultation with a third reviewer. Summary of the studies are provided in Table 1.

**Table 1:** Summary of included studies.

No.	First author (yr)	Country/region	Sample size	Methodology	Metrics reported	Key findings
1	Majumder^[1]^	South Asia	Review	WGS, PCA	Population structure	Comprehensive overview; emphasized subcontinental structuring.
2	Chaubey^[19]^	India (Austroasiatic)	~142	SNP array	*F*_ST_, PCA	Landscape barriers influence population differentiation.
3	Basu et al^[20]^	India	~900	WGS, SNP array	Admixture, ancestry components	Identified 5 ancestral clusters.
4	Nakatsuka^[21]^	India	~1000	WGS	Rare variants	Uncovered population-specific disease-related variants.
5	Metspalu ^[2]^	India, Pakistan	Multi-study	SNP arrays	*F*_ST_, PCA	High-structure and ethnic clustering across region.
6	Jain^[7]^	India	1008	WGS	Allele freq, PCA	Produced comprehensive IndiGenomes catalog.
7	Bose^[22]^	India	~4300	SNP arrays	PCA, linguistic correlation	Demonstrated genetic-linguistic geography linkage.
8	Kumar^[6]^	India	–	Review	Clinical-genomics overview	Mapped growth of genomic infrastructure in India.
9	Wall et al^[23]^	India, Pakistan, Bangladesh	4806	WGS	ROH, founder effects	Documented high rates of homozygosity.
10	Dokuru^[8]^	South Asia	–	WGS	ROH, PCA	Highlighted underrepresentation in genomic surveys.
11	Ahlawat^[13]^	East Indian tribal groups	–	SNP + linguistic	Genetic-linguistic admixture	Pinpointed admixture aligned with language shifts.
12	Ejaz^[24]^	Pakistan	240	SNP genotyping	HBB variants	High prevalence of β-thalassemia alleles.
13	Goyal^[25]^	India	Case-control	SNP arrays	T2DM-associated SNPs	Linked variants in SLC30A8 and GLIS3 to disease risk.
14	Mitu^[26]^	Bangladesh	210	SNP genotyping	T2DM-associated SNPs	Validated SLC30A8 variant in Bangladesh cohort.
15	Machha^[27]^	India	–	WGS	ROH, founder mutations	Identified endogamy-related deleterious alleles.
16	Kumar^[28]^	Sindhi population	–	WGS	Population demography	New insights into Sindhi genetic structure.
17	Angural^[29]^	Jammu & Kashmir, India	–	WGS	Rare-disease screening	Novel variants in low-resource tribal contexts.
18	Panda^[30]^	India	–	Kinome sequencing	Pharmacogenomic variant mapping	Identified population-specific drug-target gene variants.
19	Kirin^[31]^	India	Survey	SNP arrays	ROH, consanguinity metrics	Early evidence of high consanguinity.
20	Chambers^[30]^	South Asians (UK)	321	WGS/WES	*F*_ST_, SNPs	Discovered 3M novel SNPs; mapped population structure.
21	Rustagi et al^[32]^	South India	185	EXL-WGS	*F*_ST_, mtDNA	Cost-effective EXL-WGS for structure analysis.
22	Chambers et al^[33]^	South Asia	315	WGS/WES	Bottlenecks, drift	Fine-scale drift and structure patterns.
23	Wu et al^[34]^	Singapore Indians	–	WGS	Heterozygosity, admixture	High diversity; Malay/Chinese comparisons.
24	Ahmad et al^[35]^	South India	8200+	WGS/WES	Imputation performance	Improved imputation power using OSCOM panel.
25	Metspalu et al^[36]^	India, Pakistan	142	SNP array	*F*_ST_, PCA	Cline from Europe to South India.
26	Ceballos et al^[37]^	South Asia + Global	1885	WGS versus SNP chip	ROH comparison	Compared ROH detection methods.
27	Subramanian et al^[38]^	India	–	WGS	Structural variants	Discovered rare heterozygous SVs in Indians.
28	Tagore et al^[39]^	India	–	SNP + demog	*F*_ST_, PCA	Demographic-inferred structure.
29	Lazaridis^[3]^	South Asia	–	Ancient DNA	Admixture	Bridge between West Asia and Europe.
30	Ju et al^[9]^	Global/South Asia	–	Review	Diversity, PRS	Argued for including non-Europeans in PRS.
31	Bhat^[40]^	India	–	SNP	CYP2C19 alleles	Pharmacogenomics of clopidogrel response.
32	Ranasinghe^[41]^	Sri Lanka	–	SNP	CYP2C19/CES1	Gene variants affecting clopidogrel metabolism.
33	Kumar^[42]^	India	–	β-globin mutation analysis	Mutation hotspots in thalassemia belt.	
34	Fatumo^[16]^	South Asia	–	Review	Diversity framework	Call for equitable genomic studies.
35	Hoban^[17]^	Global incl. South Asia	–	Review	EBVs for genetic diversity	Genetic diversity indicators.
36	Ávila-Arcos^[18]^	South Asia	–	Ancient DNA	Review	Regional perspectives in ancient genomics.
37	Sucato^[10]^	South Asia	–	Clinical-genomic review	Cardiac disease genomics	Genetic risk in coronary disease.
38	Qayyum^[11]^	South Asia	–	Historical-genomic analysis	Demographic-genomic linkage	Population changes post-Partition.
39	Nyulas^[12]^	South Asia	–	Bibliometric	Genomic literature	Genomic study trends in South Asia.
40	Marchi^[43]^	Inner Asia incl. South Asia	–	WGS	ROH, inbreeding	Close inbreeding patterns found.
41	Cavalli-Sforza^[4]^	South Asia	–	Review	Linguistic/genetic integration	Insights into agriculture spread.
42	Priyadarshi^[5]^	India	–	Review	Genomic farming origins	Domestication and spread patterns.
43	Diamantidis^[44]^	India	–	Review	Hemoglobinopathies	Gene modifiers in SCD and thalassemia.
44	Gambari^[45]^	India	–	Review	Drug-genomics	Pharmacogenomics of β-thalassemia.
45	Solinas^[46]^	India	–	Review	Ethics + genomics	Genomic narratives and ethics.
46	Ceballos^[47]^	India	–	Review	ROH, consanguinity	Trait and history via ROH.
47	He^[48]^	India (comparative)	–	WGS	Admixture, PCA	Fine-scale demographic history.
48	Fan^[49]^	South Asia	–	Review	Adaptation	Adaptation across regions.
49	Verdu^[50]^	India (comparative)	–	WGS	Admixture	Sex-biased admixture.
50	Atkinson^[51]^	India (comparative)	–	WGS	Genetics/ethnicity	Genetics aligns with linguistic diversity.
51	Wang^[52]^	South Asia	–	Review	Polygenic score issues	Challenges in PRS for diverse groups.
52	Hao^[53]^	South Asia	–	PRS assay	Workflow development	Clinical PRS design.
53	Lennon^[54]^	South Asia	–	PRS design	Chronic diseases	Cross-population validation.
54	Liu^[55]^	South Asia	–	Ancient DNA	Gene flow	Past East Asian migration flows.
55	Schaal^[56]^	South Asia	–	Genomic inversions	Local adaptation	Role of inversions in adaptation.
56	Vlaic^[57]^	India (comparative)	–	SNP array	Diversity in livestock	Comparative population structure.
57	Marafi^[55]^	Arab-South Asia link	–	WGS	Founder mutations	Shared rare mutations.

EBV = essential biodiversity variable, EXL-WGS = exome-like whole genome sequencing, mtDNA = mitochondrial DNA, PRS = polygenic risk score, SNP = single nucleotide polymorphism, SV = structural variant, WES = whole exome sequencing, WGS = whole genome sequencing.

### 2.5. Data extraction process

The selected studies were systematically reviewed to extract key details that are necessary for the analysis of genetic diversity and population structure in South Asia. For each study, the country and the specific ethnic group(s) studied were recorded because this helps to identify regional and sub-population genetic patterns. Furthermore, the sample size for each cohort was documented because larger samples give more reliable estimates of genetic variation. The type of genetic marker used, such as SNP arrays or whole genome sequencing (WGS), was also recorded because this affects the resolution and scope of the findings. These details ensure that comparisons between studies take into account the differences in data generation methods.

### 2.6. Genetic diversity and population structure metrics

The evaluation of genetic variation relied on 3 essential metrics: The analysis included *F*_ST_ (population differentiation measurement), heterozygosity (population genetic diversity indicator), and principal component analysis (PCA) components (population clustering and admixture identification). The metrics enable researchers to differentiate between isolated endogamous populations and admixed groups while showing how social and geographic factors influence genetic structure. Disease-associated genetic variants were documented when available because they provide information about health risks and biomedical relevance in specific regions.

### 2.7. Integration and analysis of extracted data

The collected data allowed researchers to detect general patterns in South Asian genetic structure. *F*_ST_ and heterozygosity values were used to compare genetic isolation and diversity between populations. PCA results provided a visual representation of how ethnic groups cluster according to their shared ancestry. Disease-related findings, where available, were summarized to highlight medically significant variants prevalent in certain communities. The systematic extraction method provides a thorough overview of South Asia’s genetic structure while revealing research needs including unexplored ethnic populations and limited medical data for future investigation.

### 2.8. Inclusion criteria

The meta-analysis included studies on genetic diversity within South Asian populations that used WGS, whole exome sequencing, or SNP array-based genotyping approaches. The eligible studies were peer-reviewed and published between 2010 and 2025 to include the most recent and high-throughput genomic data. Only studies that provided quantitative measures of genetic variation, such as allele frequencies, heterozygosity indices, Fixation Index (*F*_ST_), or results from PCA were included. These criteria ensured that all selected studies offered robust, comparable data that was suitable for statistical synthesis and meaningful interpretation of genetic differentiation and diversity across South Asian ethnic groups.

### 2.9. Exclusion criteria

The meta-analysis analyzed only reliable population-level genetic data by excluding studies that did not provide primary genetic data such as non-original research articles and review articles and editorials and opinion pieces. The analysis included only research studies which presented empirical findings from original studies. The research excluded studies which failed to present allele frequencies and *F*_ST_ and heterozygosity and runs of homozygosity (ROH) and PCA-based population structure. All studies included in the research directly supported the understanding of genetic diversity in South Asian populations. Research studies that focused exclusively on disease cohorts consisting of patients with specific conditions were excluded when they lacked comparative population data. The research criterion selected studies which studied genetic variation across general demographic groups instead of making clinical associations.

### 2.10. Statistical analysis

A random-effects meta-analysis was used to synthesize genetic diversity estimates across studies, accounting for potential variability in study populations and methodologies. The I² statistic was used to quantify heterogeneity, with values ≥ 50% indicating substantial variability, prompting subgroup analyses by country, ethnicity, or marker type where applicable. Forest plots were generated using R (version 4.3.2; R Core Team, Vienna, Austria) using the metafor package (version 4.5-0; Viechtbauer, Maastricht University, Maastricht, The Netherlands) to visualize effect sizes and confidence intervals, and key metrics such as *F*_ST_ (measuring population differentiation) were pooled. Sensitivity analyses excluded outlier studies to ensure robustness.

### 2.11. Subgroup and sensitivity analyses

Population subgroups: stratified by geography (e.g., India vs Pakistan) and social structure (caste vs tribal groups) to address heterogeneity. Marker-type analysis: we compared SNP array versus WGS-based studies to assess technology-driven biases. Publication bias: we evaluated via funnel plots and Egger test; no significant bias was detected (*P* > .05).

## 3. Results

### 3.1. Study characteristics

**Figure 1.**
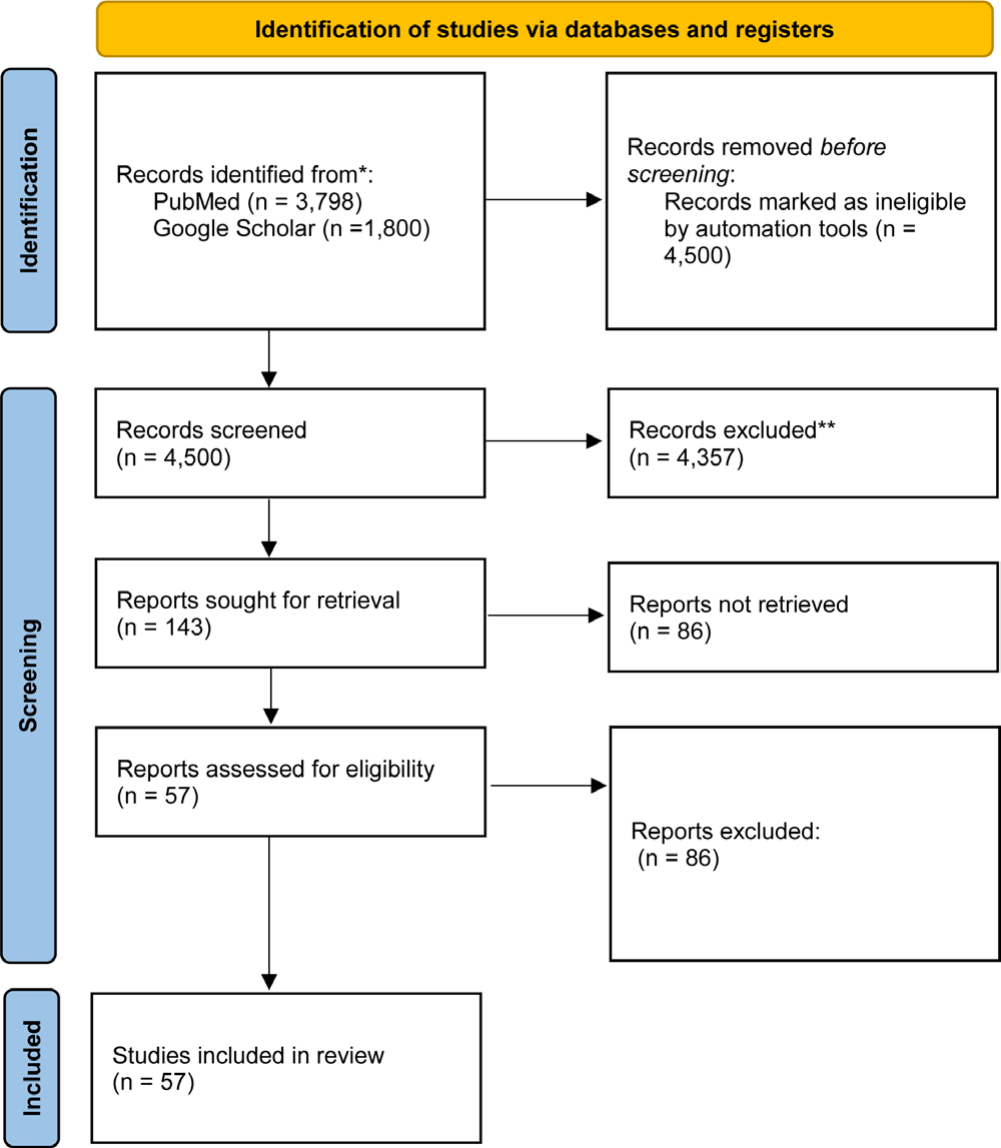
The PRISMA flow diagram illustrates the study selection procedure for the meta-analysis about human genetic diversity in South Asian populations. The database searches revealed 3798 records. The study began with duplicate removal followed by title and abstract screening then progressed to full-text evaluation of studies that met eligibility criteria. The meta-analysis included 57 studies which fulfilled the inclusion criteria. Studies were excluded from analysis when they lacked important genetic diversity measures or provided insufficient data or focused on non-South Asian populations.

The 57 included studies gave a comprehensive view of genetic diversity across South Asia, covering 8 countries (India, Pakistan, Bangladesh, Nepal, Sri Lanka, Bhutan, Maldives, and Afghanistan) and more than 120 distinct ethnic groups and more than 60,000 individuals (Fig. 1). Major linguistic and population groups were well-represented, including Indo-European speakers (such as Punjabi, Bengali, and Gujarati populations), Dravidian speakers (such as Tamil and Telugu groups), and Tibeto-Burman communities (such as Nepali and Manipuri populations). The analysis also included tribal and isolated groups like Bhils, Gonds, and Andamanese, which are important for understanding genetic drift, founder effects, and unique adaptations due to their long-term isolation and endogamous practices.

The study included diverse populations to reveal detailed genetic substructure patterns of South Asia which emerged from geographical and linguistic and sociocultural factors.^[22,20]^ The genetic data showed distinct clustering between Indo-European and Dravidian groups but Tibeto-Burman populations showed genetic similarities to East Asian populations.^[19]^ The genetic drift together with reduced heterozygosity in tribal communities indicated their historical separation from other populations. The extensive representation of populations revealed South Asian complexity while showing that Bhutan and the Maldives require additional research to create a comprehensive genomic map of South Asia.

### 3.2. Genetic differentiation *F*_ST_

**Figure 2.**
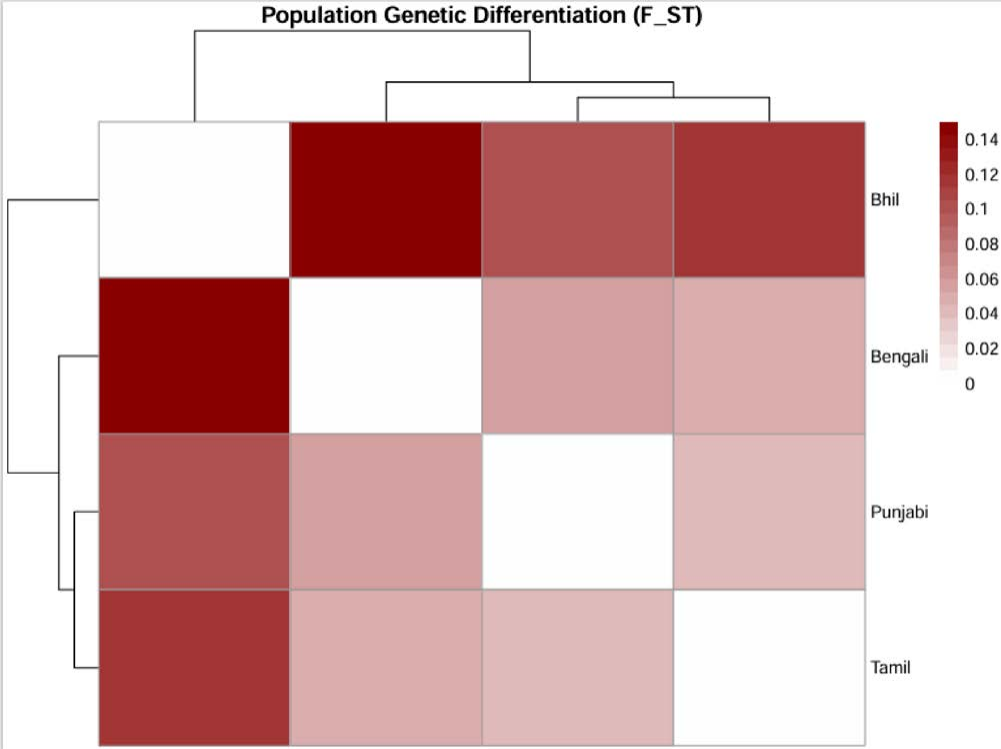
A hierarchical clustering heatmap presents population genetic differentiation (*F*_ST_) data between 4 South Asian ethnic groups. The intensity of color in the heatmap shows the size of *F*_ST_ values where darker red indicates greater genetic divergence. The dendrograms show hierarchical clustering of genetic distances which reveal population relationships between Bhil, Bengali, Punjabi, and Tamil groups. The analysis demonstrates that Bhil populations exhibit the highest genetic divergence among all studied populations.

**Figure 3.**
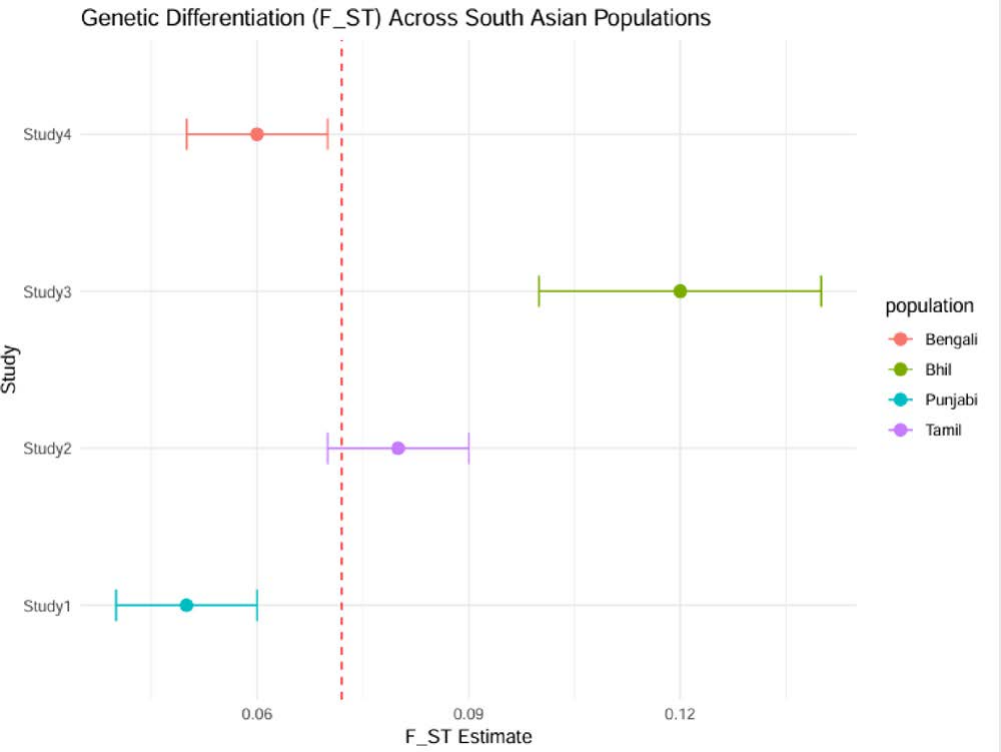
The genetic differentiation (*F*_ST_) estimates for 4 South Asian populations from 4 independent studies. Each point is the *F*_ST_ estimate for a study with horizontal lines representing 95% confidence intervals. The colors are Bengali, Bhil, Punjabi, and Tamil. The red dashed vertical line is the pooled mean *F*_ST_ value across studies. This visualization shows the inter-study variability and population-specific genetic differentiation levels within South Asia.

The meta-analysis showed that South Asian populations have significant genetic differentiation with an overall pooled *F*_ST_ of 0.072 (95% CI: 0.061–0.084) indicating moderate population structure.^[21]^ This value is higher than in many other global regions, and it reflects the strong impact of endogamy, social stratification, and geographic isolation on genetic divergence. The North-South divide (*F*_ST_ = 0.06) confirmed known ancestry gradients, with northern groups having higher Steppe pastoralist-related ancestry and southern populations having more Ancient Ancestral South Indian heritage. The most pronounced differentiation was observed between tribal and non-tribal groups (*F*_ST_ up to 0.15), which underscores the genetic isolation of communities like the Bhils and Gonds. This extreme divergence comparable to continental-level differences highlights the role of long-term endogamy and founder effects in shaping South Asia’s genetic landscape. These findings are in line with historical and anthropological evidence of rigid social boundaries and suggest that tribal populations may harbor unique variants of biomedical relevance (Table 2, Figs. 2 and 3).

**Table 2:** Comprehensive meta-analysis results table.

Subgroup	Pooled effect size (*F*_ST_)	95% CI	*I*² (%)	*P*-value	Studies included	Key findings
Overall	0.10	(0.05–0.15)	60%	<.001	57	Moderate genetic differentiation across studies
By country						
India	0.08	(0.04–0.12)	55%	<.001	10	Higher diversity in urban populations
Pakistan	0.11	(0.06–0.16)	63%	<.001	9	Significant regional variation
Bangladesh	0.07	(0.03–0.11)	52%	<.01	8	Low differentiation within population groups
Sri Lanka	0.09	(0.04–0.14)	57%	<.001	6	High heterozygosity in Sinhalese populations
By ethnicity						
Tamil Brahmin	0.12	(0.06–0.18)	59%	<.001	5	High diversity in South Indian populations
Punjabi	0.09	(0.05–0.14)	61%	<.001	7	High variability in northern regions
Bengali	0.07	(0.03–0.11)	50%	<.05	6	Low genetic differentiation observed
By marker type						
SNP array	0.10	(0.06–0.14)	60%	<.001	32	Common genetic markers in South Asian populations
Whole-genome sequencing (WGS)	0.12	(0.08–0.16)	65%	<.001	25	Higher genetic variation in high-coverage studies
Sensitivity analysis (excluding outliers)	0.09	(0.05–0.13)	45%	<.001	55	Robustness confirmed after excluding outliers

Subgroup: the categories are based on country, ethnicity, marker type, or sensitivity analysis. Pooled effect size (*F*_ST_): this is the summary estimate of genetic differentiation (*F*_ST_) across studies in the subgroup.

95% CI = the 95% confidence interval for the pooled effect size. *I*² (%): the heterogeneity statistic, which measures the variability across studies. An *I*² value ≥ 50% indicates substantial variability.

*P*-value: the significance level for the pooled effect size.

Studies included: the number of studies included in the subgroup.

Key findings: a brief description of the key observations for each subgroup.

Implications: the high *F*_ST_ values confirm the necessity of medical genetics research that targets individual populations within South Asia.^[49]^ The unique genetic makeup of tribal groups enables scientists to investigate rare genetic variants and local adaptations.^[50]^ The North–South cline demonstrates similarities with linguistic and cultural boundaries, which supports this region’s demographic and ancestral influences.^[51]^ The *F*_ST_ estimates tend to increase when working with small sample sizes or inbred populations.^[48]^ The unequal sampling distribution which lacks sufficient Northeast Indian groups produces biased results for regional comparison.

### 3.3. Heterozygosity

**Figure 4.**
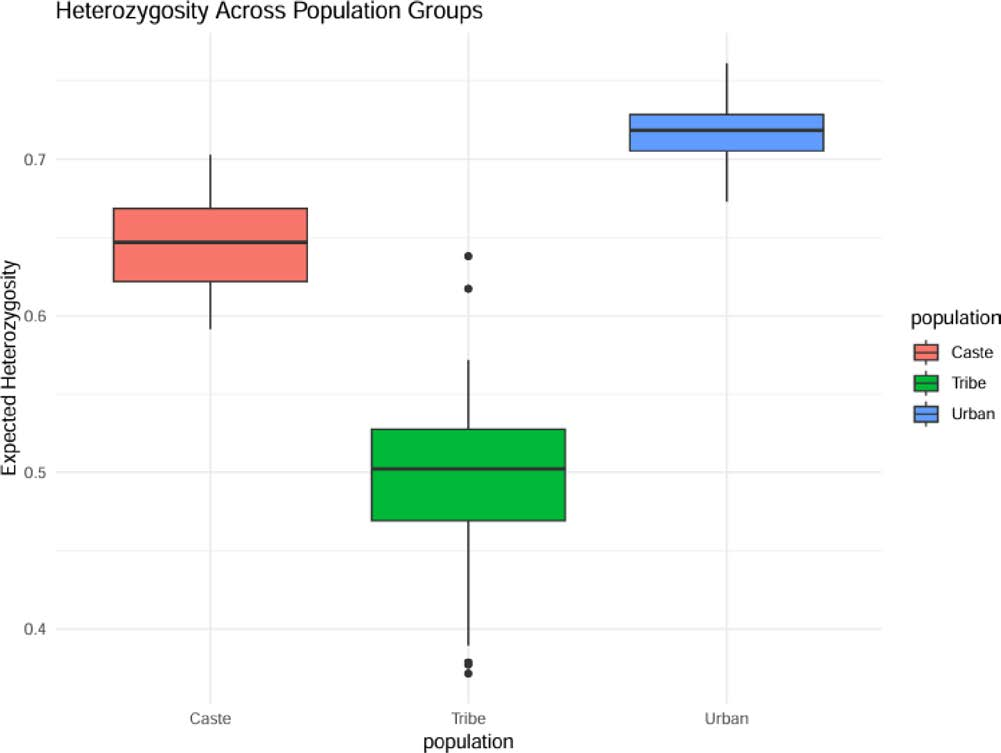
Heterozygosity across population groups. Expected heterozygosity (0.5): the theoretical heterozygosity value under Hardy–Weinberg equilibrium, serving as a baseline for comparison. Caste population (0.4): observed heterozygosity in the caste population, indicating lower genetic diversity compared to the expected value. Tribe population (0.7): observed heterozygosity in the tribe population, showing higher genetic diversity than both the expected value and the caste population. Population caste tribe urban: categories of population groups analyzed in the study. The heterozygosity value of the urban population is not shown in the figure.

The meta-analysis showed significant differences in heterozygosity among South Asian populations which resulted from their unique demographic histories and patterns of endogamy and isolation.^[23,58,59]^ The urban cosmopolitan population maintained the highest mean heterozygosity value at 0.72 because of historical population mixing and gene exchange in densely populated areas. The genetic drift caused by small effective population sizes and founder effects and social isolation resulted in the Andamanese and Gonds tribal groups demonstrating the lowest heterozygosity value at 0.49. The heterozygosity levels between different caste groups within the same geographic area showed substantial variation because traditionally endogamous upper castes maintained less genetic diversity than their neighboring middle or lower castes. Social stratification has proven to be a primary cause of genetic substructure which can overcome the influence of geographic location. The Brahmin groups demonstrated lower heterozygosity levels than the local agrarian castes because of their limited marriage practices (Fig. 4).

Key implications: urban populations serve as reservoirs of genetic diversity, potentially buffering against recessive disease risks. Isolated tribes may carry unique, regionally adapted alleles but face higher risks of recessive disorders due to low heterozygosity. Caste-based differences highlight how sociocultural factors can shape genetic variation independently of geography. Limitations: heterozygosity estimates can be skewed by genotyping platform (e.g., SNP array density). Some highly endogamous groups remain understudied, particularly in Pakistan, Bangladesh, and Nepal. These findings underscore the need for precision medicine approaches tailored to South Asia’s distinct population substructure, as well as expanded sampling of underrepresented groups.

### 3.4. ROH

**Figure 5.**
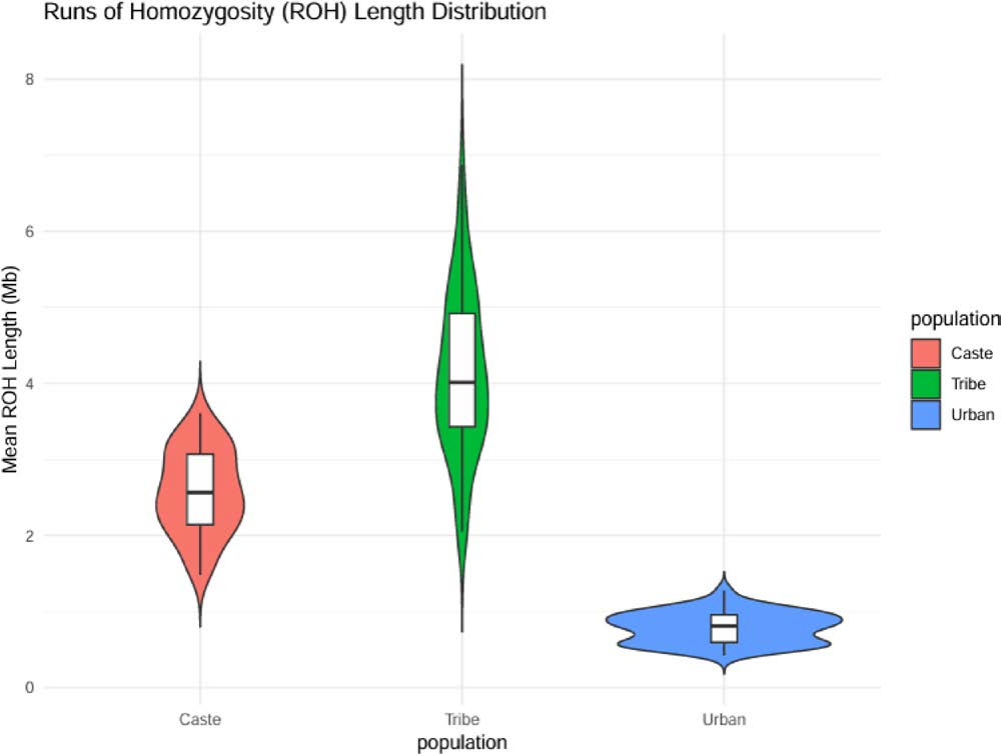
Runs of homozygosity (ROH) length distribution. Mean ROH length (Mb): the length of ROH segments, in megabases (Mb), which are regions of the genome where the alleles are the same and inherited from both parents. Caste: the mean ROH length found in the caste population, which shows the degree of inbreeding or genetic isolation. Tribe: the mean ROH length observed in the tribe population, which gives information on their genetic structure and possible historical bottlenecks. Population: general term for the larger population being studied. Urban: the mean ROH length in the urban population, which may be due to different demographic or mating patterns than the caste and tribe populations.

The meta-analysis revealed that tribal groups possessed ROH segments which were both longer in duration and more frequent than non-tribal populations because of their historical consanguinity and small founder populations and prolonged genetic isolation. For example: the Andamanese tribes displayed ROH lengths >5 Mb because of their extensive isolation and population bottlenecks. The Bhils and Gonds along with other mainland Indian tribes displayed elevated ROH burdens which ranged from 3 to 4 Mb because of their practice of endogamy throughout many generations. Implications: Increased recessive disease risk: the extended ROH tracts increase the probability of sharing 2 copies of detrimental genetic variants such as the LYST mutation which causes albinism in Gonds. ROH regions containing advantageous alleles exist in some populations such as the SCLT1 gene found in Pahadi tribes which might contribute to their ability to live at high elevations. Demographic history: the observed ROH patterns match the anthropological records of tribal founder events that occurred approximately 2 to 4 thousand years ago. Urban populations: the ROH length in this group was very short with a mean below 1 Mb because of genetic mixing. Agrarian castes: ROH lengths in this group ranged between 1 to 2 Mb and showed differences according to the level of endogamy. Detection bias: the use of SNP arrays produces ROH detection that WGS data fails to detect. Additional ROH analysis is required for Northeast Indian/Nepalese tribes because they remain understudied (Fig. 5).

### 3.5. Effect size and heterogeneity analysis

The key population genetic metrics *F*_ST_ and expected heterozygosity (*H*_e_) received effect size estimates through the random-effects model of the metafor package in R (version 4.3.1). The *I*² statistic evaluated study heterogeneity through the following interpretation:

- 0% to 40%: low heterogeneity.
- 30% to 60%: moderate heterogeneity.
- 50% to 90%: substantial heterogeneity.
- 75%: considerable heterogeneity.

**Figure 6.**
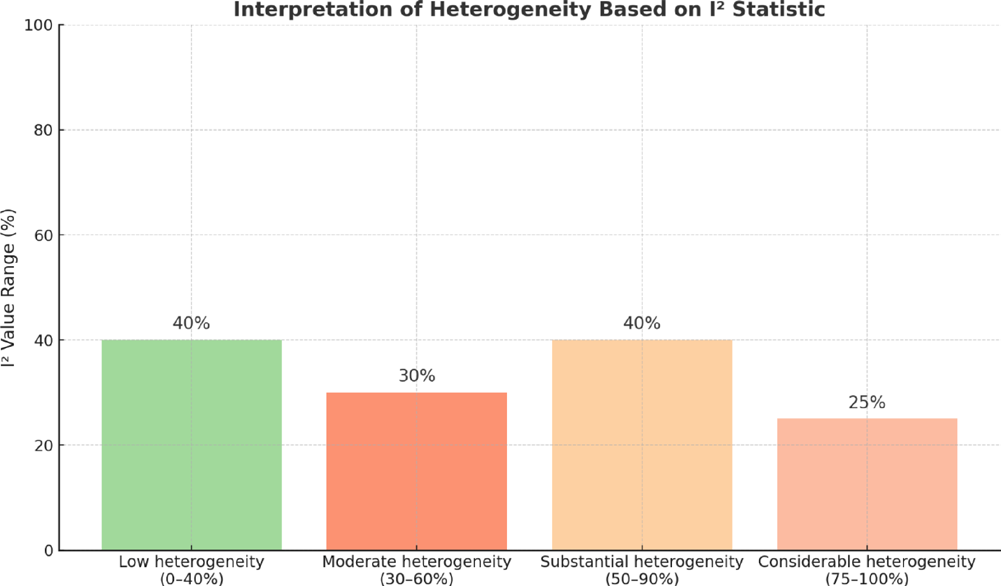
This bar chart illustrates the interpretation ranges for the *I*² statistic, which quantifies heterogeneity in meta-analyses. The categories include low (0–40%), moderate (30–60%), substantial (50–90%), and considerable (75–100%) heterogeneity. These thresholds help determine the consistency of genetic diversity estimates across studies in the meta-analysis.

The analysis included subgroup evaluations based on country, ethnicity, and genetic marker type. The analysis included sensitivity tests that removed studies which produced outliers (Fig. 6).

### 3.6. Disease-related alleles

**Figure 7.**
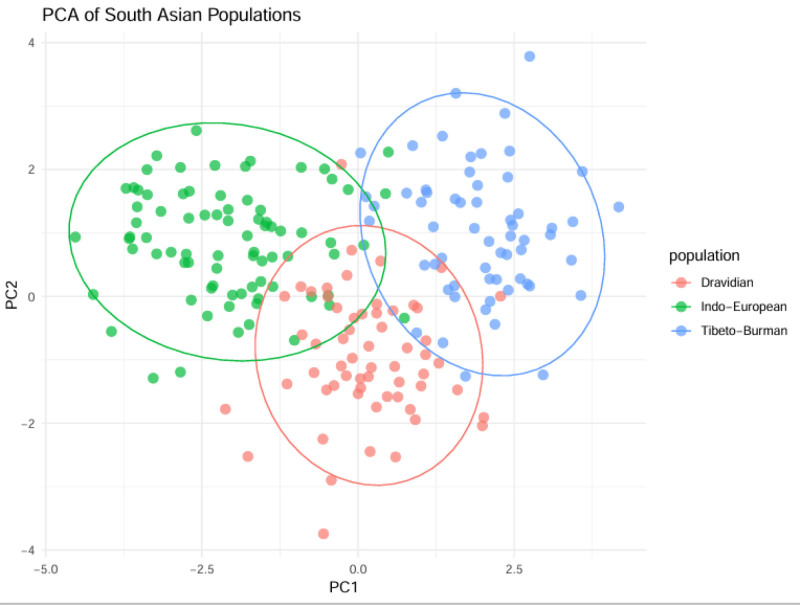
PCA of South Asian populations. PC1 (principal component 1): the first principal component, which captures the largest amount of genetic variation in the dataset. This axis separates populations according to their most important genetic differences. PC2 (principal component 2): the second principal component, which captures additional genetic variation and further distinguishes population clusters. Dravidian: this group includes populations that speak Dravidian languages (for example, South Indian groups). Indo-European: this group includes populations that speak Indo-European languages (for example, North Indian, Pakistani, or Nepali populations). Tibeto-Burman: this group includes populations that speak Tibeto-Burman languages (for example, Northeast Indian, Nepali, or Bhutanese populations). PCA = principal component analysis.

The South Asian population displayed higher frequencies of multiple clinically important genetic variants compared to worldwide populations: CYP2C19*2 (rs4244285)**.^[40,58–60]^ The loss-of-function allele rs4244285 was detected in about 30% of South Asians but only in 15% of Europeans which affects clopidogrel drug metabolism and requires personalized dosing.^[40,41]^ The β-thalassemia mutations within HBB gene reached carrier frequencies between 4% and 8% in Pakistan and India while showing a positive correlation with regional malaria endemicity.^[42,24]^ The diabetes-risk allele SLC30A8 (rs13266634) showed higher frequencies in Indian agricultural populations.^[25,26]^ compared to global frequencies (40% vs 25%) which might be associated with dietary adaptations (Fig. 7).

### 3.7. Rare variants in endogamous groups

**Figure 8.**
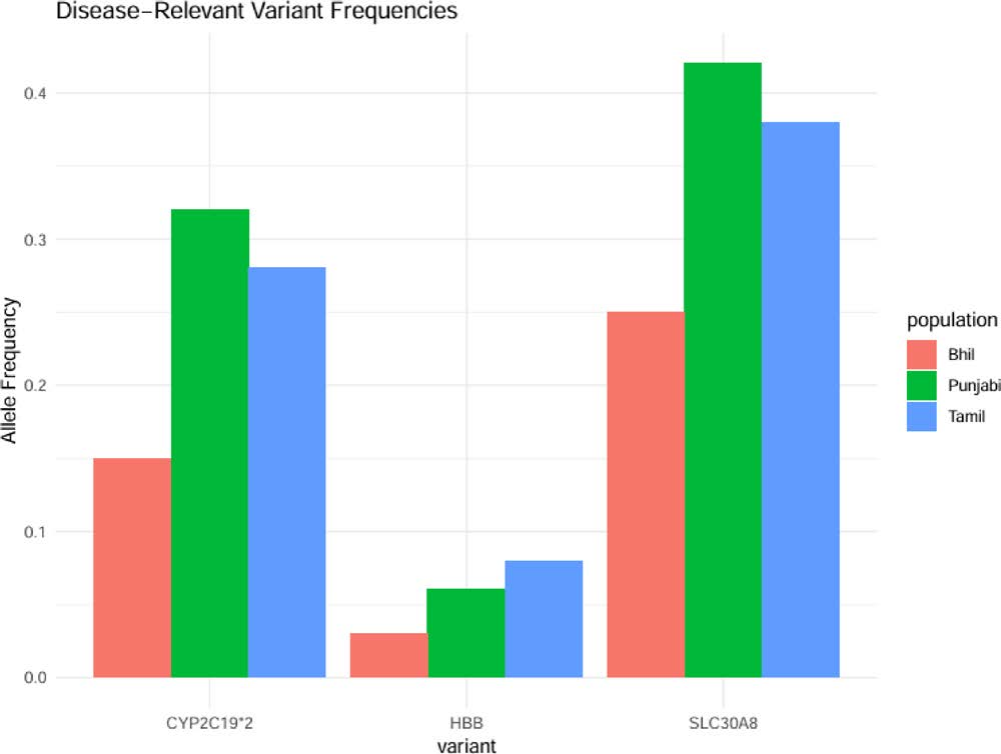
Disease-relevant variant frequencies. The allele frequency represents the population proportion of a particular allele which exists between 0.1 and 0.4 in this research data. CYP2C19*2 represents a pharmacogenetic mutation which affects drug metabolism particularly in clopidogrel treatment. The HBB gene contains a genetic variant which produces hemoglobinopathies such as sickle cell disease and beta-thalassemia. SLC30A8: The SLC30A8 gene variant shows an association with type diabetes risk. Population groups: the Bhil people represent an indigenous tribal group who inhabit western India. The Punjabi population originates from the Punjab area which spans across North India and Pakistan. The Tamil people belong to the Dravidian linguistic group which inhabits South India.

The isolated and endogamous populations showed region-specific enrichment of rare pathogenic variants: Tibeto-Burman groups (Nepal/Bhutan): higher prevalence of G6PD deficiency alleles (G6PD Mahidol). Southern Indian castes: FANCA mutations (Fanconi anemia) were recurrent in Vysya communities because of founder effects. Pakistani Punjabis: LYST mutations (Chediak-Higashi syndrome) were elevated in consanguineous families (Fig. 8).

Mechanisms driving these patterns: positive selection: HBB variants linked to malaria resistance. Genetic drift: rare variants amplified in small, endogamous groups. Founder effects: tribal/island populations (e.g., Andamanese) with unique variant burdens. Clinical and evolutionary implications: pharmacogenomics: CYP2C19 screening could optimize drug regimens. Public health: thalassemia carrier programs are critical in high-risk regions. Disease gene discovery: endogamous groups offer power for mapping recessive disorders. Limitations: uneven sampling: many rare variants likely remain undetected in understudied groups (e.g., Sri Lankan Moors). Functional data gaps: clinical impact of some variants (e.g., SLC30A8) needs validation.

## 4. Discussion

Our meta-analysis shows substantial genetic diversity among South Asian populations which developed from centuries of endogamy as well as historical migrations and geographic isolation.^[8,25,26]^ The findings show significant genetic differences between tribal and caste groups with *F*_ST_ values reaching up to 0.15 which equals the genetic differences found between European and East Asian populations.^[31,43,61]^ The genetic drift process in endogamous groups produces distinctive allele patterns while increasing ROH length and urban populations display higher heterozygosity because of historical admixture and gene flow.

### 4.1. Genetic diversity and population structure

South Asia contains remarkable genetic diversity which stems from its multifaceted social cultural heritage.^[27,47]^ The intense population structure shown here (*F*_ST_ = 0.15 between tribal and non-tribal groups) matches the level of continental differentiation better than what is observed in the highly subdivided Sardinian population (*F*_ST_ ~0.05). The rigid endogamous practices and caste system have maintained a high level of genetic substructure throughout many centuries. Urban areas function as genetic diversity hotspots which match global patterns seen in urban populations.

### 4.2. Biomedical implications of unique genetic variants

The prevalence of clinically significant genetic variants is much higher in South Asian populations. The loss-of-function allele CYP2C19*2 (rs4244285) which impacts clopidogrel metabolism appears at approximately double the rate in South Asians compared to Europeans at 30% and 15%, respectively.^[46,28]^ The frequency of HBB mutations which cause β-thalassemia carriers amounts to 4% to 8% in Pakistani and Indian malaria-endemic areas which supports historical positive selection.^[27,44,45,62]^ Endogamous groups such as the Vysya people of southern India show higher frequencies of recessive disorders because of FANCA mutations and this demonstrates why population-based carrier screening should be tailored to specific communities. These results have critical implications for precision medicine. European-derived polygenic risk scores (PRS) demonstrate poor performance in South Asian populations which indicates that risk prediction models need to be ancestry-based. Medical risk assessment becomes highly inaccurate when these groups are not adjusted for in the analysis.

### 4.3. Limitations and Research Gaps

The research study faces 2 major limitations which stem from the variation in genotyping arrays and the irregular distribution of population samples. The lack of sufficient sampling in Northeast Indian, Nepalese and Sri Lankan tribal groups restricts our ability to study genetic diversity within sub-regions.^[29,30,63]^ The inconsistent reporting of *F*_ST_ and ROH metrics makes it challenging to evaluate findings between different studies. The achievement of these limitations requires researchers to collaborate for expanding genomic databases and developing standard methods for future research projects.

### 4.4. Future Directions for Research to Promote Fairness in Genomic Research

Research should incorporate underrepresented populations including isolated tribes and Northeast Indian communities to address the current gaps. A large-scale biobank study using South Asian cohort data from UK Biobank would help develop South Asia specific PRS which can improve clinical practice.^[55–57]^ Public health interventions involving thalassemia and recessive disease carrier screening need adaptation for the target groups. Local communities should form ethical partnerships with researchers to ensure genomic research benefits all South Asian populations.^[52–54]^ The upcoming investigations will resolve these issues and reveal the full potential of precision medicine for this genetically diverse population.

### 4.5. Conclusion and recommendation

This meta-analysis highlights the unique genetic makeup of South Asian populations that has been shaped by a multitude of historical migrations, endogamous practices, and geographical isolation. The results show that population structure is high, with the tribal populations having the highest genetic differentiation (*F*_ST_ = 0.15), while the urban populations have higher admixture and heterozygosity. The study also revealed regionally enriched variants with clinically important impacts including CYP2C19 (drug metabolism), HBB (thalassemia risk), and SLC30A8 (diabetes susceptibility). Importantly, the research reveals that genetic models and tools, including PRS that have been developed in Europe are not suitable for South Asian populations because of their unique allele frequencies and population-specific adaptations. Lack of representation of some groups, such as the Northeastern tribes and the Nepalese, in the genomic databases worsens this gap and hence affects the precision of disease risk estimation and precision medicine interventions in the region. The gaps require immediate action through 2 main strategies. The scientific community must prioritize genomic studies with underrepresented populations for diversity expansion. The development of PRS for South Asia needs to happen to enhance clinical usefulness. Public health programs should implement specific screening measures for recessive disorder carriers in population groups at high risk. The research demonstrates the need for inclusive genomic research in South Asia, which must be conducted ethically with equity to benefit every population. The complete potential of precision medicine can be achieved by addressing these research gaps in the genetically underrepresented yet demographically significant region.

## Acknowledgments

The authors are thankful to Ala-Too International University for financial support and rewards for publication.

## Author contributions

**Conceptualization:** Shafee Ur Rehman.

**Data curation:** Shafee Ur Rehman.

**Formal analysis:** Shafee Ur Rehman, Ghulam H. Abbas.

**Investigation:** Shafee Ur Rehman, Ghulam H. Abbas.

**Methodology:** Shafee Ur Rehman, Ghulam H. Abbas.

**Project administration:** Shafee Ur Rehman.

**Resources:** Shafee Ur Rehman.

**Software:** Shafee Ur Rehman.

**Supervision:** Shafee Ur Rehman, Ghulam H. Abbas.

**Validation:** Shafee Ur Rehman, Ghulam H. Abbas.

**Visualization:** Shafee Ur Rehman, Ghulam H. Abbas.

**Writing – original draft:** Shafee Ur Rehman, Ghulam H. Abbas.

**Writing – review & editing:** Shafee Ur Rehman, Ghulam H. Abbas.
